
JOURNAL INFORMATION
==============================
NLM Title Abbreviation: Northwest Med Surg J
NLM Journal ID: 0020727

The North-Western Medical and Surgical Journal

Publisher: Biomedical Journal Digitization Project

ARTICLE INFORMATION
==============================
PMCID: PMC9970585
PMID: 37320684
Article version: 1
Subjects: Miscellaneous Items

Cholera in Moscow

Print publication date: 1856 Oct
Volume: 5
Volume Series: New Ser
Issue: 10
First page: 485
Last page: 485
License: This work is free of known copyright restrictions. For more information, please see PMC Back Issue Digitization.
License URL: https://creativecommons.org/publicdomain/mark/1.0/

==============================

*Cholera in Moscow.*

Cholera is raging fearfully in Moscow, which is said to be the true cause of the postponement of the coronation of the Czar.

---

### STARLING MEDICAL COLLEGE, COLUMBUS, OHIO.

The TENTH ANNUAL COURSE OF LECTURES in this Institution, will commence on the FIFTEENTH OF OCTOBER, and continue full five months.

**FACULTY.**

| | |
|---|---|
| S. M. SMITH, M.D., | Professor of Theory and Practice, and Dean. |
| F. CARTER, M.D., | Professor of Obstetrics and Diseases of Women and Children. |
| JOHN DAWSON, M.D., | Professor of Anatomy and Physiology. |
| J. W. HAMILTON, M.D., | Professor of Surgery. |
| THEO. G. WORMLY, M.D., | Professor of Chemistry. |
| S. LOVING, M.D., | Professor of Mat. Med. and Med. Jurisprudence. |
| R. N. BARR, M.D., | Demonstrator of Anatomy. |

TERMS:

Lectures, $60. Matriculation, $3. Graduation, $20.
Board, Lodging, &c., $2 50 to $3 per week.

By a law, passed at the late session of the Legislature, Students of this Institution are admitted free to the Hospital of the Ohio Penitentiary, thus furnishing one of the best Clinics in the country.

For further information, address S. M. SMITH, DEAN
